# *Campylobacter*, 3rd edition

**DOI:** 10.3201/eid1412.081165

**Published:** 2008-12

**Authors:** Sean F. Altekruse

**Affiliations:** National Institutes of Health, Rockville, Maryland, USA

This valuable new edition documents advances in *Campylobacter* spp. molecular biology, epidemiology, immunology, and food safety interventions over the past quarter century. It is an easy-to-read resource for both lay readers and scientists who are active in these research fields.

The book contains 6 complementary sections. The first section provides an overview of the taxonomy of *Campylobacter* and related species, followed by in-depth chapters on population biology and molecular techniques. The second section reviews clinical and epidemiologic aspects of human infections. The next 3 sections describe advances in pathogenesis, immunity, glycobiology, and gene expression. Food safety interventions are discussed in the final section.

Throughout the book, current references are cited, such as one that describes a recent finding of a possible link between *C.* *rectus* and *C*. *concisus* infections and Barrett’s esophagitis, a precursor of esophageal adenocarcinoma. One chapter provides an up-to-date summary of risk factors associated with human campylobacteriosis. Other chapters describe current methods for examining antibiotic resistance, mechanisms of resistance, and subtyping infectious strains. The center of the book is dedicated to technical research areas that may eventually lead to new prevention technologies. The final chapters relate to policies for enhancing food safety.

The third edition of this book is a valuable reference. It provides a history of the science for this field, a review of current understanding, and expert guidance on avenues for future research. Its theme can be summarized by paraphrasing Stephen On and colleagues: With the integration of statistics, epidemiology, biology, and a risk-based paradigm *Campylobacter,* science is coming of age. Future discoveries are likely to be as thrilling, challenging, and surprising as the developments of the past 25 years (On et al., Chapter 10, p. 207).

